# Role of the arts in the life and mental health of young people that participate in artistic organizations in Colombia: a qualitative study

**DOI:** 10.1186/s12888-022-04396-y

**Published:** 2022-12-03

**Authors:** Carlos Gómez-Restrepo, Natalia Godoy Casasbuenas, Natalia Ortiz-Hernández, Victoria Jane Bird, María Paula Jassir Acosta, José Miguel Uribe Restrepo, Bryan Alexander Murillo Sarmiento, Mariana Steffen, Stefan Priebe

**Affiliations:** 1 Departament of Clinical Epidemiology and Biostatistics, Pontificia Universidad Javeriana, Bogotá, Colombia; 2 Departament of Psychiatry and Mental Health, Pontificia Universidad Javeriana, Bogotá, Colombia; 3 San Ignacio University Hospital, Bogotá, Colombia; 4 Unit for Social and Community Psychiatry, WHO Collaborating Centre for Mental Health Services Development, Queen Mary University of London, London, UK; 5 People’s Palace Projects, Queen Mary University of London, London, UK

**Keywords:** Mental health, Adolescence, Art, COVID-19, Depression, Anxiety, Resilience

## Abstract

**Background:**

Adolescents and young adults are vulnerable to developing mental distress. However, evidence suggests that more than half of the young people with symptoms of depression and anxiety overcome their distress within a year. However, there is little research on the exact resources that young people use and help them to recover. The aim of this study was to explore how arts activities can support the recovery of young people engaged with arts organizations in Bogota.

**Methods:**

We recruited 38 participants from two arts organizations in Bogotá and conducted six focus groups embedded within artistic workshops. The type of activities in the workshops varied reflecting the different teaching methods of the two organizations. The focus group discussions were recorded and analyzed using thematic analysis.

**Results:**

Five themes explained how arts activities can help young people participating in artistic organizations to overcome mental distress: i) allowing the expression of emotions; ii) helping to manage and transform emotions; iii) distracting from problems; iv) facilitating social support and relationships; and v) contributing to the identity of young people.

**Conclusions:**

For young people who participate in artistic organizations, the arts are a resource for overcoming negative emotions such as anxiety, depression, and sadness. The beneficial role of arts activities includes different process of managing, expressing, and distracting from distress, and it differs depending on whether arts are perceived as a professional vocation or a hobby.

**Supplementary Information:**

The online version contains supplementary material available at 10.1186/s12888-022-04396-y.

## Introduction

According to a 2016 report, adolescents and young adults in Latin America and the Caribbean region make up approximately 26%, of the general population [1]. Even though efforts have been made towards improving poverty and economic inequality in Latin America, it remains the region with highest socioeconomic differences, a factor that is known to have a negative effect on the life and health conditions of the population [1, 2]. Evidence suggests that factors, such as socioeconomic status, quality of life at home and relationships with others, predispose young people and adolescents towards an increased risk of developing mental disorders [2, 3].

Adolescence and early adulthood are moments within the life cycle which represent important changes for individuals [4, 5]. During this period of time, adolescents and young people encounter different challenges and social changes, whilst attempting to transition successfully into adulthood [5]. Changes in various aspects of life, including physical, psychological and sociocultural features [4], may make young people particularly vulnerable to mental health problems [1]. In Colombia, barriers to access formal educational, experience or fear of violence, early start of sex life, extensive urban migration and lack of work opportunities are common risk factors among young people for violence and delinquency, use of psychoactive substances, undesired pregnancy and mental health problems [6].

The World Health Organization have reported that approximately 10–20% of young people have undiagnosed or untreated mental health problems [3]. Moreover, it is estimated that by the year 2030, depression will be the leading cause of disease burden globally [7]. Evidence suggests that having a mental health problem during adolescence is a risk factor for having other psychiatric problems later in life [8, 9]. |Around 75% of mental health illnesses begin before the age of 25 [10]. Thus, the mental health of young people should be considered a public health priority [9].

According to the 2015 National Mental Health Survey, in Colombia, the lifetime prevalence of depression or anxiety in adolescents is 2.4 and 5.0% respectively [11]. This compares to adults, where the lifetime prevalence of these disorders are 5.3% for depression and 3.9% for anxiety [11]. The present COVID-19 pandemic can be considered an exacerbating factor in relation to the mental health of young people [12]. According to a survey conducted by UNICEF in Latin America and the Caribbean region, 27% of young people reported symptoms of anxiety and 15% of depression during the pandemic [13]. A survey conducted in Bogota, found that 32% of the participants reported feeling more anxious or nervous as compared to before the lockdown, whilst 27% of participants stated to feel significantly more anxious or nervous after the lockdown [14]. However, in general, between 50 and 60% of young adults and adolescents who manifest symptoms of depression and anxiety do not develop longer-term mental disorders and are able to overcome these episodes of emotional distress within 1 year after the onset of symptoms [15]. This process of bouncing back from symptoms is captured in the concept of resilience [15].

Resilience is a broad term describing responses to the experience of adversity. It can be understood in different ways [16–18] Resilience can be seen as a personal characteristic of individuals that enables them to recover from distress. However, resilience can also signify the process of recovering itself, i.e., something that individuals or groups show when experiencing distress. In this article, we use a wide understanding of the concept of resilience, regarding it as the process of bouncing back from episodes of mental distress, but also include the abilities and qualities that help to overcome adversity and distress [18].

One important aspect of resilience are the personal and social resources that affected people use and help them to overcome distress [19]. Some resources are intrinsic to the individual, whilst others are considered external [19]. The external resources relate to social and cultural aspects in the environment, like family, interpersonal relationships or participation in activities, such as the arts [20, 21]. Some evidence suggests an association between specific protective factors, such as family attachment, social skills, strong moral beliefs, positive personal disposition, among others, and the reduction of mental health problems [22].

In relation to the arts, there has been increasing evidence showing an association between participation in arts activities and positive health. Various reports and reviews in the medical literature have mentioned the benefits of artistic activities for the mental health of individuals and community well-being [23]. In young people, engaging in these types of activities has been associated with positive outcomes such as the development of social skills and positive behavioral changes [21]. Participation in arts activities has also been suggested to increase self-esteem, feelings of achievement, empowerment, and social skills and to promote socializing with others [21]; all of these characteristics can be seen as related to the concept of resilience [21]. However, the existing studies and the available evidence in this area are inconsistent and have mainly originated from European countries, meaning that the evidence for the mental health benefits of arts activities of young people participating in arts organizations within a Latin American context, specifically Colombia, is limited.

## Methods

### Study design

This study is part of a multicentre study called OLA (Building resilience and resources to reduce depression and anxiety in young people from urban neighborhoods in Latin America). This study is conducted simultaneously in three capital cities in Latin American countries (Bogota, Lima, and Buenos Aires).

Within the first phase of the study, we conducted focus groups that were embedded in artistic workshops. The workshops were by arts organizations that promote and teach different types of artistic expressions, like singing, drawing, and musical composition. The workshops were intended to engage young people and create a conducive atmosphere for the discussion in the focus groups. This article reports the analysis of the data collected in Bogotá, Colombia.

### Objectives

The main objective of this study was to characterize the role of the arts in relation to the mental health of young people who are involved in different arts organizations in Bogota. The main objective of the artistic workshops and focus groups was to explore the experiences of overcoming episodes of emotional distress in young people, including but not exclusively focusing on artistic activities. The participants were recruited from two arts organizations in Bogota: Familia Ayara and Batuta Foundation.

### Arts organizations

Batuta Foundation is an organization whose main aim is to improve the quality of life of children, adolescents and young people in Colombia through musical education that focuses on collective practice from a social inclusion, rights and a culturally diversity perspective [24].

Familia Ayara organization is an institution inspired by the 1980’s New York Hip Hop movement. Through the different types of Hip Hop artistic expressions, Familia Ayara seeks to generate spaces where young people can express themselves, generate dialogue and reflect on the problems that affects them [25].

These two organizations helped in the development of the workshops by implementing different artistic methods as used in their own practice. The artistic workshops explored the experience of depression and anxiety and the role of the arts for the recovery from emotional distress. They were also intended to engage participants and help them to open up and share experiences in the following focus group.

### Participants

A convenience sampling method was used to recruit participants. This was facilitated by the coordinators of the arts organizations associated with the project, taking into account the interest of the young people who were active in their organizations. Each arts organization coordinator called students who had a history of emotional distress and invited them to participate in the artistic workshops and subsequent focus groups. The potential participants were contacted by phone and email. Approximately, 20 students per organization were contacted and the ones who were available at the time and signed the informed consent form were able to participate in the workshops.

The inclusion criteria were being 15–24 years old, wanting to participate in artistic activities, being currently active in the activities provided by the arts organization, having the capacity to consent (for those aged18 and over), receiving consent from a parent or legal guardian (for those aged 17 and under) and have capacity to assent (for those aged 17 and under).

#### Data collection

##### Artistic workshops

In total, six artistic workshops were conducted: four with Batuta Foundation and two with Familia Ayara. The workshops were designed to provide the context and set the scene for the focus group discussion. They facilitated engagement, allowing for and utilizing non-verbal communication and artistic expression, helped to establish trust and created an open atmosphere for the participants. The workshops were adjusted to the approaches of the two the organizations. Nevertheless, there were common features. Each workshop had two phases: the first part consisted of ice breakers, explaining the context of the workshop and the use of different artistic activities for self-expression; the second part were a structured conversation within a type of focus group, initiated by questions for the participants delivered by a moderator. During the second phase of each workshop, the conversation was guided towards identifying the characteristics, resources and activities participants had used to overcome moments of emotional distress. The moderators for these conversations were part of the OLA research team and mental health professionals (psychiatrists) at the Pontificia Universidad Javeriana. On average, the workshops lasted between 2 and 4 h and there was only one moderator per focus group.

Taking into account that the first phase of the OLA study took place in 2020, the artistic workshops were adapted to a virtual modality in order to comply with the social distancing requirements of the Colombian government due to the COVID-19 pandemic. These workshops were held virtually through the Microsoft Teams platform.

*Batuta Foundation: Artistic workshop description*

The four workshops conducted with the Batuta Foundation had a total of 20 participants split into 4 groups (five participants per group). The workshops were facilitated by members from both, the OLA research team and Batuta Foundation, the latter were present to assistant with the structure, guidance and execution of the workshops, particularly in relation to the artistic activities held.

The artistic activities held during the workshops are described below:

- Introduction round: The participants introduced themselves and were asked to perform a body movement that would identify them.
- Sounding Scenery: The participants had to relate sounds that were played to images displayed for them.
- Learning song lyrics: With the help of one of the organization teachers, the participants repeated the lyrics to the song “Yenyeré Gumá” whilst the instructor sang simultaneously.
- Changing the lyrics of known songs: The song “Color Esperanza” by Diego Torres was played and the participants had to listen and then jointly write new lyrics for the melody of the song. The lyrics had to be in relation to the words mentioned after they were asked *“What has music meant for you in your life?”*.
- After each activity, the participants provided a word describing their feelings during the activity.

*Familia Ayara: Artistic workshop description*

The workshops conducted with Familia Ayara had a total of 17 participants, who were divided in two groups. Reflecting the variety of artistic activities that this institution provides, the workshops had different approaches towards the arts. One of them included activities related to the rap musical genre and the other one with graffiti art. During these workshops members of the research team were present, as well as a teacher and a member of the psychosocial department of the organization. These last two individuals helped in guiding the workshop.

i. *Rap Workshop:*

During this workshop the following activities were held:

- Introduction round: The participants presented themselves through a rap improvisation.
- Rap composition and socialization: The participants chose one of three music tracks to work with and composed rap lines with 8 beats each. The themes of the rap lines related to premises of self-recognition, emotions, life projects and stress management. After the composition period, the participants were asked to share their compositions.
- The participants provided a word describing how they felt before and during the workshop.

ii. *Graffiti Workshop:*

During this workshop the following activities were held:

- Introduction round: The participants, members of the organization and research team introduced themselves.
- Word transformation through drawing: The participants were asked to think of a word that represented a negative emotion they felt during the pandemic and quarantine. The participants were then asked to write this word using separate strokes so that the lines that formed the words did not touch. Lastly, they were asked to transform each line into a positive image through a drawing.

*Focus group discussions with the participants*

During the second phase of each artistic workshop, conversations were held with the participants in relation to the role of the arts in their lives, their mental health, and the resources they used to overcome episodes of emotional distress. The objectives of these conversations were to explore the representations of depression and anxiety using artistic methodologies; to identify and describe physical, affective, cognitive, behavioral, and social manifestations of depression and anxiety according to their experience; and to describe the main causes of emotional suffering. These conversations lasted between 60 and 80 minutes.

#### Data analysis

The focus group discussions were recorded in audio format, transcribed, and analyzed using an inductive thematic analysis methodology proposed by Braun and Clarke [26]. Three researchers familiarized themselves with the transcriptions and from the reviewed information generated a coding and thematic scheme that reflected the role of the arts within the participants’ lives and its relation to their mental health. The themes and subthemes were discussed with the research team and modified iteratively. During this process, an initial coding scheme was developed. It was finalized following team review and discussion. Afterwards, two research team members applied the coding scheme to the transcriptions using NVivo 12® qualitative software.

After coding all the transcriptions, a report that describes the content of each code was generated and this was the basis for the result synthesis. Finally, codes were discussed as a group and then grouped into broader categories that yielded the themes reported in this paper.

#### Ethics

All methods and instruments used during this process were revised and approved by the research and ethics committee of Queens Mary, University in London, and by the research and ethics committee of San Ignacio University Hospital of Pontificia Universidad Javeriana. The aforementioned follows the established protocols of the Good Clinical Practice guidelines according to the Colombian legislation. The analysis plan was elaborated and approved by the principal investigators. None of the focus groups were corrected or repeated. The data as collected during August and September of the year 2020.

## Results

A summary of the demographic characteristics of the participants in relation to their age range, sex and the artistic workshop they attended can be found in Table 1.

**Table 1:** Characterization of the participants by age, sex, and artistic workshop

Arts Organization	Total Number of Participants per Arts Organization	Age Range	Number of Participants per Age Range	Gender	
				Male	Female
**Familia Ayara**	17	**15–19**	7	2	5
		**20–24**	10	7	3
**Batuta Foundation**	20	**15–19**	16	5	11
		**20–24**	4	2	2

Five themes were identified from the workshops: 1) Mobilizing emotions and facilitating their expression; 2) Help to manage or transform difficult emotions; 3) Distracting from problems; 4) Creating spaces for social support and facilitate social relations; 5) Contributing to the construction of identity and lifestyle. The five themes are shown in Table 2 where the themes correspond to the first row and each column resumes the subthemes used in each theme. As this study was conducted during the COVID-19 pandemic, within the results a section alluding to the emotional experiences of the participants during this period was included.

**Table 2:** Coding scheme

THEMES	SUBTHEMES
**Allowing the expression of emotions**	Allows to express and vent emotions
	Generates or arouses different emotions or feelings
**Helping to manage and transform emotions**	Help transform emotions and negatively perceived thought
	Gives tranquility and stability
**Distracting from problems**	Allows to forget or distract from problems
**Facilitating social support and relationships**	Artistic groups bring social support
	Facilitates communication and connection with others
**Contributing to the identity of young people**	Encourages self confidence
	Are a component of a person’s identity and lifestyle
	Help to orient and give life meaning
	Are a source of professional projection

### Allowing the expression of emotions

One of the most frequent themes that emerged about the role of artistic activities in relation to mental health, is the ability to communicate emotions and thoughts through arts. Several young people mentioned that artistic expressions allowed them to vent or unload their worries or negative thoughts. In some cases, it helped them to overcome certain difficulties they had in recognizing and verbally expressing their feelings. Others take advantage of writing, musical composition, or painting as a means of understanding and clarifying difficult situations, particularly when they are unwilling to talk about their problems with friends or family, due to mistrust or fear of judgemental responses. Additionally, some young people have found Hip Hop a vehicle to express and show their views and thoughts about their lives and the reality of the country.

*“I think it is a way of expressing oneself. Then each one can transmit or express, as my colleague said before, something that one feels personally. Then, from that, you can vent, either on a wall, or on a piece of paper. And so, one is a little freer to express what one feels through art, or through something that one practices.” (Familia Ayara, F, 19 years old).*

*“I feel that I would lose a lot of stability, uh, on an emotional level. I feel that it is the only thing that keeps me in order and, let’s say, healthy, right? Speaking of this topic, which is art. It’s being able to express certain things. I’ve never been good at talking, like at expressing my emotions to others through conversation. I almost can’t do it, so I think music has helped me a lot in that. Art in general.” (Batuta Foundation, F, 18 years old).*

*“This is like my strong suit, my refuge. Singing too. I have many revolutionary lyrics, in disagreement with what is happening here in Colombia [...] So rap also helps me to vent the frustrations I have, and to make my voice heard.” (Familia Ayara, M, 21 years).*

Some of the Batuta Foundation participants mentioned that musical interpretation has the potential to generate different emotions in them that they would not usually experience in other contexts. Depending on the song they perform, they may feel emotions of joy, happiness, tranquility, nostalgia, or sadness. Similarly, the mood they are in influences the graphic creations of those who practice painting or graffiti.

*“For me art is a way to feel a lot of things that I can’t feel like in my day-to-day life, yes? And also, I see art as a refuge. And actually, it wasn’t like I knew about it and I that’s what got me into art, but one day I just tried it and I really liked it and I realized like all the things I can feel, and I didn’t know, so that was very important for me.” (Batuta Foundation, F, 18 years old).*

### Helping to manage and transform emotions

Some participants report that artistic expression helped them manage stress, depression and anxiety. Through art, they also found tranquility and a way to transform intense emotions of anger and negative thoughts. Others alluded to the usefulness of the arts in managing these emotions during the COVID-19 pandemic.

*“When, for example, I feel angry with myself or sad, then that’s how I react with the instrument. Let’s say I’m going to play something and let’s say I put like more... I’m angry, I put like more pressure on things, to make it sound harder. So, let’s say that for me it’s a way of... like of channeling those sensations and, when transmitting them, let’s say that I play for myself. Then, at that moment, I play something and ten minutes, twenty minutes can go by and after that moment it’s... let’s say it’s as if I’ve let go of all that tension I had, it’s a way of like taking off that pressure I have.” (Batuta Foundation, M, 21 years old).*

*“Not only drawing, not only listening to music, not only writing, but everything that has to do with art. I feel that it liberates me a lot. It kind of leaves me at peace, it calms me down a lot, in the face of all those situations that are sometimes so complicated. So, it’s like the psychologist you have, to whom you can tell how you feel. Without thinking about others and that’s what I like so much about this.” (Familia Ayara, M, 22 years old).*

### Distracting from problems

Some participants claimed that attending music workshops or playing an instrument helps them to distract themselves, forget problems and difficulties. According to their description, through artistic activities, distraction seems to encompass multiple psychological aspects described as “having a more active mind”, “putting the mind in blank”, “getting away from reality”, or “feeling in another world”.

*“Well, I take my board and go out on it. I put big wheels on it, I go out with my bag, with my posters, which are for signing, two or three aerosols, a white background. And then I go out rolling. The truth is I don’t know where I’m going to paint, where I’m going to end up. (...) I go out with my board, I end up in places I don’t know, over there climbing on a terrace, painting a board, painting a fence. Well, that distracts me a lot, and it’s also like the adrenaline of not getting caught by the police”. (Familia Ayara, M, 21 years old).*

*“Well, I think that music separates us... it takes us away from reality and we start, like... I don’t know how to explain it... I don’t know... I mean, it makes us feel like we are in another world, yes? we feel better and all that, like so.” (Batuta Foundation, F, 15 years old).*

### Facilitating social support and relationships

The participants highlighted the opportunities offered by arts organizations to explore their interests or discover artistic activities and languages. They have also found in them different forms of social support. Familia Ayara provided a space for young people with shared interests to meet, which has helped individuals to overcome feelings of being “out of place”. Those who are part of Batuta Foundation consider it an important space to meet other people and create bonds of friendship with their peers. Teachers, facilitators, and psychosocial support staff also represent an important support for some of the participants.

*“When I’m not feeling well, I go and talk to the assistant and the psychosocial worker. And in spite of that, in the 8 years that I have been at Batuta, I have formed bonds of friendship. I have seen people come and go, and I could always count on them, but some of them left and others never came back. And well, that’s how it has helped me: to learn that people are not always there, but if you look for them, I believe they will be there”. (Batuta, M, 17 years old).*

Some participants involved with Batuta Foundation see music as a way to connect with others. They say that the ease of expression they develop through musical performance has helped them to communicate, especially those who find it difficult to socialize. Others note that music has facilitated a cohesion with their fellow orchestra members.

*“If art did not exist, I think there would be a lack of union between human beings, because through art one can..., as you were saying, a harmony is generated, or a connection with other people. Or, for example, in my musical experience, while you make music you connect...”. (Batuta, F, 18 years old).*

### Contributing to the identity of young people

Rapping and playing a musical instrument has also helped some participants to get to know themselves better, as well as to recognize their strengths and weaknesses. In some cases, facing an audience has allowed them to gain confidence.

*“Well, before learning about music, I was confident about playing sports [...]. And after music... I learned to express myself with the microphone, how to handle the public and what one does. Now, what you write and... It has to give you power, because if you don’t believe it, others won’t believe it. So, I think that’s one of the bases of the self-confidence that I have worked on”. (Familia Ayara, M, 22 years old).*

Artistic expression has also been important in the identity construction for some young people, especially among those who participate in graffiti art and Hip-Hop culture through Familia Ayara. They describe these practices as a central element in their daily lives. They also relate them to the possibility of finding direction or a plan for their lives. Some highlight the opportunity Familia Ayara offers to keep them busy and away from criminal activities and drug use. In addition, the support they receive from this organization has motivated some of them to project themselves professionally as artists.

*“I used to be a very quiet person [...] I had a lot of trouble expressing myself. Hip Hop has helped me a lot with that, to express myself, to vent. And Hip Hop means everything to me. I mean, it’s my life, it’s the way I express myself, the way I think, it’s my lifestyle. Uh... I want to make a professional living from music. For me that means that it’s... It was what saved me from myself, what gives me calm, what makes me feel, really. That’s what Hip Hop means to me.” (Familia Ayara, M, 22 years old).*

### Emotional implications of the COVID-19 pandemic

When probed about how they have experienced the pandemic, the lockdown periods, and the possible effects this has had on their mental health, some participants mentioned that this led to feelings of insecurity and frustration, usually related to the difficulty of making plans or trusting that their projects will come to fruition.

*“I think the first thing it has taught us since the pandemic started is that nothing is certain, no... no jobs, not what you want to do, not the plans to leave. As they say, the only thing that is certain is that we are going to die, nothing in this life is certain, plans can change from one day to the next.” (Familia Ayara, M, 22 years old).*

One participant recounted how painting allowed her to work out her uncertainty about leaving her job just prior to the onset of the pandemic.

*“After three days they said that no one could leave, everyone was in confinement. I was left with my arms crossed and I said, what am I going to do now? where am I going to get a job? So, I said, well, while something comes up, let’s wait. And well, I took paper and watercolors and in reality, I wasn’t feeling good, but I wanted to use the time. I felt that, well, in the first brushstrokes when I had already done the face, I said: I don’t know, it’s not reflecting what I want. So, I had a fixed point and I said: no, I want it to reflect sadness, because that’s how I feel [...] So, let’s say that it is something satisfying when you do something with a purpose, with an end and it has the expected results.” (Familia Ayara, F, 17 years old).*

Additionally, some participants had to get used to a decrease in their academic, work and leisure activities. Although several participants continued to take part in artistic activities, others put them on hold. In the case of the Batuta Foundation, the participants did not have access to the musical instruments provided by the organization, whilst as a result of the mobility restrictions established during the pandemic some participants linked to Familia Ayara limited their outings to graffiti related activities. Other participants considered that this change was positive, as they reduced the activities that caused them stress, such as the use of public transport or the fulfillment of schedules and obligations, which allowed them to dedicate more time to activities of interest and pleasure.

*“At the beginning of my quarantine it was difficult for me to stay here at home because I was busy every day of the week. Monday through Friday I would go to school, Saturdays I would do some activity and on Sundays another one. So, to be in constant movement and then to stop, technically, because of the pandemic, well, it was difficult. But now [...] I am more used to being in four walls.” (Batuta Foundation, M, 17 years old).*

## Discussion

The findings suggest that arts can be an important resource for young people who participate in arts organizations. They show different ways for how arts activities can help young people to cope with mental and emotional distress and how arts organizations can provide psychological and social support. Despite the differences between the institutions and their artistic methodologies, we found similarities in the mechanisms that the participants identified in relation to arts and the mental health benefits arts activities provide. Engaging in artistic activities promotes resilience in young people and allows them to bounce back from symptoms of emotional distress.

### Emotion management and its relationship with the arts

We found that the arts are useful for the mobilization and expression of emotions, particularly when the emotions are perceived as negative, and they also help young people to recognize and manifest how they feel. The results suggest that not all artistic activities evoke the same emotions, and that this depends on the type of artistic activity being performed. For example, writing seems to serve a more internal purpose, related to understanding emotions, while other activities, such as musical performance, graffiti, painting, or rapping, contribute to externalizing thoughts and feelings.

Artistic expression can also help to manage psychological stress and states of anger, depression, and anxiety, since it provides a way to transform them. Although the recognition or venting of emotions and their transformation seem to be strongly linked, these aspects were analyzed in different themes. The study did not intend to address and analyze differences between the psychological process of recognizing and relieving emotions or transforming them. Nevertheless, the findings could relate to the evidence on emotion regulation through the arts, as they suggest that arts activities may support emotion regulation in individuals with depression [27], and that negative emotions decrease and positive emotions increase when engaging in art-based group activities [28].

In relation to the transformation of emotions, within the Familia Ayara workshops the creativity was evidenced through the graffiti activity. In this activity, the participants made a drawing that narrated a significant story, starting with a word that reflected a negative emotion drawn with separate strokes, with the purpose of changing the meaning of the word. This process leads not only to creativity and expression of the individual’s emotional state, but also to the reconstruction of situations and emotions for the individual. This technique of combining the writing of words and transforming them into drawings is reminiscent of the technique described by Winnicott, in which the patient elaborates a trace that culminates in a figure or vice versa, giving a story understood within the personal history of the individual [29]. This has also been reported to be effective in the context of child therapy [30, 31].

Participants stated that artistic activities, such as attending music workshops or playing an instrument, are a source of distraction and help them forget about problems or difficulties. Distraction seems to be a multidimensional theme, distraction occurs in relation to other aspects of the arts, such as pleasure and the meditative nature of the arts, among others. Additionally, distraction from problems and emotional management through artistic activities are closely linked, since distraction as a result of these activities could be considered a way of managing emotions. Depending on the individual and the given context of experiencing negative emotions distraction may be more helpful, while for others it may be more useful to vent or confront their emotions. More studies are needed for a better understanding of the factors determining the most beneficial forms of dealing with emotions when using the resource of arts activities. The arts, by serving as a vehicle for emotions, could have a preventive role in the development of mental health problems in young people, or serve as a therapeutic exercise when there is difficulty in expressing negatively perceived emotions.

The findings on the link between arts and overcoming emotional problems are consistent with findings from other studies that analyze the benefits of arts activities for mental health in both adult and youth populations [21, 23, 32, 33]. Some of the benefits found in the literature include self-expression, expression of feelings, distraction from illness/problems, and a perceived improvement in mental health well-being [21, 23, 32, 33].

#### Personal and professional identity in relation to the arts

Throughout the study we found that the participants perceive their relationship with the arts differs according to the approach and vision of art taken within each organization. Several participants from the Familia Ayara see art as a lifestyle and as an integral part of their identity, and likewise perceive art as a professional vocation, rather than as a hobby or pastime. This is consistent with some of the benefits of the arts found in another study with young people, that emphasized the role of arts for perceived security, a sense of belonging, and the development of social skills [21]. This aspect of the role of arts activities was less present in the participants from the Batuta Foundation, although some of them mentioned how they identified with musical performance or considered it as a professional career option. To understand this, it should be noted that the Familia Ayara works around an artistic expression that is part of a broader cultural framework: Hip Hop. Through this, the organization not only invites young people to develop different artistic practices (rap, graffiti and break dancing) and provides them with important technical support, but they also offer active psychosocial support. This may reflect the way in which the Familia Ayara participants perceive art in their lives. However, more research is needed to explore the role of the arts in the mental health of people who perceive the arts as a career and those who use the arts as a hobby.

### COVID-19 pandemic and the arts

One aspect to consider in this study is the impact of the COVID-19 pandemic. Firstly, the impact of the pandemic has led to a substantial worsening in the mental health of young people worldwide [12]. Therefore, we can assume that some participants in this study underwent considerably long periods of social isolation and mandatory quarantine (6 months) [34] and were particularly susceptible to symptoms of depression and anxiety. Participants also mentioned that, in some cases, the restrictions imposed by the pandemic made it difficult for them to engage in artistic activities. However, for the participants who continued to actively participate in some form of art during lockdown or the self-isolation period, this may have acted as additional emotional support which prevented or mitigated symptoms of depression and/or anxiety. Finally, activities in this study allowed the participants to interact socially for the first time since confinement, which some of them described as an exciting experience.

## Strengths and limitations

To our knowledge, this is the first study to explore how the arts help young people who participate in arts organizations to cope with mental and emotional distress in a Latin American country, such as Colombia. As far as we know, this is also the first study with young people in Latin America embedding focus group discussions within artistic workshops as a part of the data collection process, allowing participants to engage using non-verbal communication and artistic expressions. A further strength of our study is the variety of artistic expressions used among participants - music composition, rap, graffiti, singing, etc. - which facilitated a deeper understanding of the different ways in which arts and arts organizations can support young people in overcoming mental and emotional distress. Even though the fact that arts can support mental health has been widely suggested in the literature, the evidence for the claim is still weak. The study adds to the literature in general as it provides more data on the experiences of young people, used an innovative methodological approach of linking artistic workshops with focus groups, and applies specifically to young populations in urban environments in Latin America.

Another strength of the study is the arts organizations’ ability to adapt and be flexible during the COVID-19 pandemic. Initially, all the artistic activities (related to music, rap, graffiti, etc.) were planned to be conducted in-person, but due to the restrictive measures implemented in Bogota, they had to be adapted and conducted on virtual platforms. Even though the virtual execution of the activities proved challenging, it was also interesting to see how each organization was creative enough to adapt their methods by using innovative strategies.

We were also able to capture some of the experiences of isolation due to the quarantines and changes in the social dynamics (family, friends, academic environment, etc.) due to the COVID-19 pandemic. A finding that was not anticipated during the original design of the study.

The study also has some limitations. Conducting focus groups and arts workshops virtually may have influenced the interaction between the facilitators and the participants, and impacted the comfort and openness to discuss certain topics. Despite this, the data collected was rich and varied, allowing us to better understand the role of arts in overcoming mental and emotional distress among young people. The results apply to young people who are involved with artistic organizations, and there is no evidence on whether arts activities may also be beneficial to those who are reluctant and would require more persuasion to participate. Finally, our findings were generated in a specific context, and it remains unclear to what extent they can be generalized or apply only to the studied age group and the context of large urban settings in Latin America such as Bogota. Additional research is needed to understand how the arts help young people cope with emotional distress in more rural settings.

## Conclusions

Study participants reported arts activities as an important resource for managing negatively perceived emotions, such as stress, anxiety, depression, and sadness. The management of these emotions can occur in different ways, because arts activities help either to transform these emotions, to vent or express them or to distract from them. However, even though these three ways of emotional management overlap, the psychological processes that are behind each one appears to be distinct and may merit specific further research.

There are differences in the perception of arts activities as a resource depending on whether they are perceived as a professional vocation or a mere hobby. This is also related to the vision instilled by the art institutions and the type of artistic disciplines they teach. Although the participants of both organizations mention art as an important part of the construction of their identity and professional vocation, this is more accentuated in the participants of Familia Ayara.

Overall, the findings of this study suggest that artistic activities in groups, such as composition, drawing, writing, music performance, and hip hop, are both a support and a resource for young people who engage in these types of activities.

## Supplementary Information

**Additional file 1.**

## Data Availability

The consent forms did not specify that the data would be deposited in a public repository, and for this reason we are unable to deposit the data. The dataset will be made available from the corresponding author (Natalia Godoy Casasbuenas, natalia.godoy@javeriana.edu.co) upon reasonable request and subject to a data sharing agreement.
