# Supplementary material for: Role of the arts in the life and mental health of young people that participate in artistic organizations in Colombia: a qualitative study
Source: BMC Psychiatry. 2022 Dec 3;22:757. doi: 10.1186/s12888-022-04396-y (PMC9719131; doi:10.1186/s12888-022-04396-y)
Supplement: Supplementary file 1 — Additional file 1. [file 12888_2022_4396_MOESM1_ESM.pdf]

OLA WP1: Topic guide for focus groups with adolescents, young adults and professionals to explore views on resources that young people use to overcome mental distress

Introduction and group rules (5 mins)

Thank participants for their availability, introduce researchers and explain:

- All participants here have signed a consent form that describes the study and the aim of the group.
- Aim of the group: to explore and understand your views on the types of resources that young people use to overcome mental and emotional health problems. By resources we mean what helps people to recover, specifically personal resources (for example, what people do and how people might cope in different situations), and social resources (for example, your friendships).
- We are interested in seeing if the resources that young people use to recover from mental and emotional health problems change as they grow older; and so we are involving young people in our study who are aged 15-16 and 20-24 years.
- Confidentiality: the names of the participants will only be known by the researchers and other participants in today's focus group, and will not be revealed to anyone else.
- The session will be audio-recorded, transcribed and analysed by researchers.
- All participants will be assigned an ID number and all potentially identifying information will be removed.
- Audio recordings will be destroyed once data analysis is complete.
- One researcher will facilitate the discussion using a topic guide. The second researcher will make sure all areas to be discussed are covered and to write notes about important points raised.

<u>Outline group rules</u>:

- Information shared within the group must remain confidential. We ask that you try not to use names if talking about services or staff members.
- Speaking one at a time and listening and respecting others' opinions
- You do not have to share information that you do not want to
- There are no right or wrong answers
- We are looking for the widest possible range of views. Alignment of views within the group is not required
- Mobile phones to be switched off or on silent mode (unless required for work/urgent personal reasons)

- Any questions?
- Start audio recorder

Ice breaker (approx. 5 mins):

Ask each participant in turn to say:

- Their name or nickname
- And something about themselves, e.g. one thing they enjoy doing or a talent they have

Focus group discussion (approx. 45 mins):

Aim: to explore and understand the views and experiences of adolescents, young adults and professionals on the types of resources that young people use, alternative ways of measuring resources and on the resources included in the assessment battery.

During adolescence and youth, it can be common for people to experience mental and emotional health problems, which includes feelings of depression, anxiety and stress. Living in big cities can mean people are more likely to experience stressful events. However, not all people develop mental and emotional health problems, and when people do become emotionally distressed, many will recover within a year. And so we want to understand what helps people to recover.

Introduction:

- What are the situations or experiences that might cause emotional distress in young people?

Types of resources:

- What helps young people to recover from mental and emotional health problems?
- What resources do young people have in their daily life that help with feelings of emotional distress?
- What helps people if they are feeling down, upset or anxious?

Prompts:
Family/partner
Friendships
Groups in community such as religious groups, youth centres
Sports activities – alone or in groups
Arts activities – alone or in groups
Internet/social media
Personal resources (thinking styles, coping strategies, self-care)
Green spaces in cities/going out of the city/contact with nature
Can you think of other things that help people?

- How do they help?
  Prompts:
  Do they help all people, or just some people?
    - Are there resources that are more helpful for young people depending on their gender?
    - What resources are more helpful for young people of male/female/non binary gender?
    - Are there resources that are more helpful for your age group compared to the younger/older age group?

- Are there resources in the local community for young people?

    - What type of resources

- How do young people learn about and access resources in the local community?
    - How do people find out about these resources?
    - Do they need a referral to start attending a group, club or organisation?
    - Do they need to pay a fee?

- What are your/young people's experiences of accessing these resources?
  Prompts:
  Was it easy to access them and why?
  Did you face any barriers or difficulties?
- What resources would young people like to see and access in the local community?

<u>Measuring resources</u>:
In this research study, we would like to understand better the resources that help young people to overcome mental and emotional health problems. To help with this, we have put together a booklet to ask young people about the resources that they use. The booklet consists of a range of questionnaires that can be completed by young people. At the moment, the resources that are included in the booklet are: (show and read through list of resources).

- In your opinions, are there resources or areas that are important for mental and emotional health to young people, which are missing from the booklet?
    - What resources?
    - Why?
    - How would you ask young people about these resources?

- Are there resources in the booklet, which you think are not relevant for young people?
    - What resources?
    - Why?

- Do you have any other thoughts or comments about the resources in the booklet?

- We currently plan to ask young people about these resources using a booklet of questionnaires. What do you think about this approach?
    - How should young people complete this booklet of questionnaires? (by themselves, with the help of a researcher?)

- What other ways do you think we could use to identify and ask about the resources that young people use, apart from this booklet?
    - Can you think of other approaches to explore resources with young people?

Conclusion (up to 10 mins)

- Does anyone have anything else to add to what has been discussed today: any further comments or recommendations about resources for recovering from mental and emotional health problems?
- Does anyone have questions for us about this study?
- Thank participants for their contribution
